# Iatrogenic Complications of Compulsory Treatment in a Patient Presenting with an Emotionally Unstable Personality Disorder and Self-Harm

**DOI:** 10.1155/2021/6615723

**Published:** 2021-05-27

**Authors:** Charlotte Burrin, Natasha Faye Daniels, Rudolf N. Cardinal, Catherine Hayhurst, David Christmas, Jorge Zimbron

**Affiliations:** ^1^University of Cambridge School of Clinical Medicine, Cambridge, UK; ^2^King's College, Cambridge, UK; ^3^Hughes Hall, Cambridge, UK; ^4^Cambridgeshire and Peterborough NHS Foundation Trust (CPFT), Cambridge, UK; ^5^Department of Psychiatry, University of Cambridge, UK; ^6^Emergency Department, Addenbrooke's Hospital, Cambridge, UK

## Abstract

Attempted suicide and deliberate self-harm are common and challenging presentations in the emergency department. A proportion of these patients refuse interventions and this presents the clinical, legal, and ethical dilemma as to whether treatment should be provided against their will. Multiple factors influence this decision. It is difficult to foresee the multitude and magnitude of complications that can arise once it has been decided to treat individuals who do not consent. This case illustrates a particularly complex chain of events that occurred after treating someone against their will who presented with self-harm and suicidal ideation. These consequences are contrasted with those of not intervening when similar situations arose with the same patient.

## 1. Background

The ICD-10 defines emotionally unstable personality disorders (EUPD) as having a marked tendency to act impulsively without consideration of the consequences, together with affective instability [1]. Attempted suicide and deliberate self-harm by people with EUPD are common presentations to the emergency department (ED) [2, 3]. A proportion of these patients refuse interventions and this presents the clinical, legal, and ethical dilemma as to whether treatment should be provided against their will [4]. Multiple factors influence decision-making in a crisis, but in the case of patients refusing treatment following significant self-injury, there is a tendency for clinicians to insist on treatment, even when the patient has the mental capacity to decide [5]. There are multiple reasons for this insistence on treatment, including fear of harm to the patient and fear of the medicolegal consequences of not treating [6, 7].

It is difficult to foresee the multitude and magnitude of complications that can arise once it has been decided to treat individuals with a personality disorder against their will. These incidents usually arise in times of distress, and the process of enforcing treatment in this group could generate a vicious cycle of further distress, generating further incidents. Compulsory treatments can include physical restraint and the compulsory administration of psychotropic drugs, both of which are associated with their own complications. Psychotropic drugs may be associated with neuroleptic malignant syndrome [8] which is characterised by fever, muscle rigidity, and altered mental status. Physical restraint in the supine position can predispose to aspiration pneumonia and thrombosis such as pulmonary embolism and can even result in restraint asphyxia [9]. Whilst compulsory treatment is associated with significant risks, one cannot discount the risks of not treating. Those who self-injure are at significant risk of suicide, with a correlation between repetitive self-harm and self-cutting and eventual suicide [10].

It has been highlighted that those suffering from EUPD utilise services more frequently [11]; therefore, there is a need to employ compassionate methods of caring for this group which reduces the risk of coercion. An escalation in frequency or severity of presentations could increase the risk of medicolegal consequences. Therefore, coercing treatment in some instances to avoid perceived medicolegal complications may be self-defeating.

This case illustrates a particularly complex chain of events that occurred after treating someone presenting with self-harm and suicidal ideation against their will. We contrast these consequences with those of not intervening when similar situations arose with the same patient.

## 2. Case Presentation

M is a 22-year-old woman with a history of EUPD. She has been known to child and adolescent mental health services (CAMHS) since the age of 14 with multiple characteristics of EUPD including impulsivity, lack of consideration of consequences of her actions, unpredictable and capricious mood, outbursts of emotion, incapacity to control behavioural explosions, quarrelsome behaviour and conflict with others, poor self-esteem, unstable interpersonal relationships, self-destructive behaviour, and suicide attempts. Her presentation to secondary mental health services was precipitated by sexual abuse by her brother when aged 10 and subsequent bullying at school due to her weight. She typically presented to the ED with extensive self-harm and suicidal intentions triggered by auditory hallucinations involving the voice of her brother. She was a psychiatric inpatient between 2011 and 2016 and responded well to a combination of clozapine and levomepromazine. She was subsequently discharged to supported accommodation.

She had 14 admissions to the local acute psychiatric unit in the calendar year before the admission discussed here. The factors influencing her presentations were considered to be multifactorial, including being the victim of a sexual assault in May 2018 and nonconcordance with medication.

In September 2018, she was brought to the ED by police under section 136 of the UK Mental Health Act with self-harm wounds and disclosing to police officers her intention to walk to a nearby motorway to commit suicide. She was extremely agitated, handcuffed to the bed trolley, and restrained for prolonged periods by four police officers and two members of the hospital security team. The police stayed in the hospital with her for nine hours, and subsequent restraint was carried out by six security staff. Staff support and various forms of sedation were used during the initial hours in ED to try and reduce her agitation and step-down restraint.

Her medication on admission was clozapine 250 mg/day, paliperidone depot 75 mg/month, sertraline 150 mg/day, pirenzepine 50 mg/day, omeprazole 20 mg/day, atenolol 50 mg/day, ferrous fumarate 420 mg/day, desogestrel 75 micrograms/day, and an etonogestrel contraceptive implant.

She had deep lacerations on her left arm and both legs, 12–14 cm in length, exposing subcutaneous fat. Surgical review concluded that the injuries required exploration and closure under general anaesthesia, but that the wounds did not pose a risk to life or limb, nor did they require emergency surgery or admission to a surgical bed.

As she was detained under section 136 of the MHA (Mental Health Act) and there was no readily available alternative place of safety, she was admitted to facilitate a psychiatric assessment. She consistently refused treatment and was threatening to complete suicide on a local dual carriageway; twice earlier that year she had been removed from walking along the carriageway of the same road.

A Mental Health Act (MHA) assessment took place 18 hours following admission. M was detained under section 3 of the MHA. She was scheduled for surgery to repair her self-harm wounds. She remained suicidal and continued trying to remove her lines and dressings.

This was the start of a 128-day admission to the acute hospital where several life-threatening iatrogenic complications arose (Table 1). M was restrained at least 17 times, and intramuscular (IM) medication was used in nine of these incidents. She was intubated and transferred to the intensive care unit (ICU) four times to manage her behaviour.

Various members of both the medical and liaison psychiatry (LP) teams involved determined on at least a daily basis that she did not have capacity to make decisions about her medical care and treatment. This opinion did not alter throughout her medical admission.

M was finally transferred to Springbank ward, a specialist personality disorder unit in Fulbourn Hospital, on the morning of her 128th day of admission. At the point of transfer to Springbank, the liaison psychiatry and medical teams considered her mental state to be similar in terms of distress, attempts to self-harm (in frequency and severity) and statements of intended suicide as those throughout her acute hospital admission, including her first presentation to the ED. At the point of discharge, she was in ICU on 2 : 1 special observations. Her mobility was limited to 80 yards with a Zimmer frame and two members of staff assisting her. She required rapid access to the toilet because of urinary and faecal urgency. She had difficulties speaking or lying flat. Her leg wounds required ongoing dressings, and she required outpatient follow-up by cardiologists, ENT surgeons, and respiratory physicians.

## 3. Springbank Admission

Springbank ward [12] is the only specialist personality disorder unit in the NHS that accepts patients detained under a section of the MHA. It offers a one-year treatment pathway for women with a personality disorder who have not benefited from acute and community services or who are still considered to be at high risk of completed suicide. The use of the MHA is avoided, and detained patients are expected to set a discharge date from their detention early on in their admission.

M was discharged from section 3 MHA after 18 days at Springbank. She was redetained under section 3 MHA on day 238 of her admission, due to a suspected psychotic episode when she was threatening to jump in front of traffic. The detention was followed by a series of severe self-harm incidents. The detention was rescinded after two days when it was established that her behaviour was not secondary to psychosis, but part of her personality disorder. When given the option of leaving the ward, she opted to stay, and her self-harm stopped.

There were 26 significant incidents during her 372-day admission to Springbank (Table 2). The severity of the incidents on the ward was of equal or greater severity than those at first presentation to the ED. She frequently refused any interventions. The general approach adopted was to ask her to stop the self-harming behaviour, ask her to remove any inserted foreign bodies (or offer help in doing so), and ask her whether she wanted any treatment. Her wounds would typically require suturing, but she would often refuse this and refuse to go to the ED. Control over and responsibility for her treatment was given to her.

She was never restrained and rapid tranquillisation was never given. She was never physically violent towards staff. The complications arising from not enforcing treatment (usually skin infections and large scars) were minimal in comparison to those that had arisen at the acute hospital and the general psychiatric ward (Table 1). Medication was not enforced. She was discharged into supported accommodation after completing the one-year treatment programme.

Eight structured outcome measures were used to monitor her progress at Springbank. M showed improvement in nearly all outcome measures (discharge versus admission; supplementary data Table 3).

On discharge, her regular medication included olanzapine depot 405 mg/fortnight, lithium carbonate 1000 mg nocte, prazosin 3 mg nocte, levomepromazine 50 mg tds, promethazine 50 mg tds, pregabalin 300 mg bd, levothyroxine 100 mcg od. *Pro re nata* (PRN) medications included levomepromazine 50–100 mg every 4 hours, diazepam 5 mg (up to 40 mg/day), salbutamol, Peptac, and codeine phosphate 30–60 mg qds.

At the time of writing (September 2020), M has been discharged for 248 days. She has not had any further hospital admissions. Supplementary data Table 4 summarises her service use before and after her admission to Springbank. She is currently living in supported accommodation, engaging in voluntary work, and has been self-harm-free for over one year.

## 4. Perspectives

Several members of staff involved with the case were interviewed for this report.

### 4.1. Emergency Department

M was brought in by police very agitated. ED staff made a quick decision to give IM sedation to try to stop the need for restraint. They were surprised that this was only partially effective and believed that more sedation was needed whilst waiting for a MHA assessment, in order to maintain her safety. M was not well known to either ED or LP staff on shift. Due to her level of sedation, she was unfortunately not admitted to the ED-led clinical decisions unit whose staff are used to nursing patients with mental health needs, but instead to a medical bed.

### 4.2. Acute Medical Ward

In the early hours of the admission, the acute medical team found it difficult to get an expert opinion in such a complex psychiatric case, which they felt was beyond their professional capability. There were difficulties contacting LP at night (input from a senior liaison psychiatrist is not provided 24 hours/day), and the duty nursing officer in the local psychiatric hospital was unable to provide advice. When the on-call psychiatrist was called, they were off-site and unable to attend as they were covering the entire region. The large doses of medication used made it difficult to prescribe anything further to manage M's behaviour. The nurses felt unable to cope with her behaviour on the ward and reported finding it very stressful and traumatic.

### 4.3. ICU

ICU staff reported that whilst dealing with psychiatric presentations in ICU is not uncommon, M was the most difficult they had faced. The use of drugs for sedation was particularly challenging, as maximal doses were required and intramuscular administration made the pharmacokinetics less predictable. Because of M's extreme behaviour, intubation was often felt to be the only option for her safety, as well as to protect staff. Whilst this was felt to be undesirable, it was seen as the only solution due to M's determination to take her own life. The iatrogenic complications that resulted were seen as unavoidable due to the potential alternative of suicide.

### 4.4. Liaison Psychiatry

LP saw themselves as the primary providers of psychiatric care in the general hospital. Their view was that once self-harm had escalated to a life-threatening level and all other behavioural and pharmacological measures had failed, formal sedation (general anaesthesia) was a necessary step during treatment, though not without significant risks. Their strategy thereafter was to seek to optimize psychotropic medications and physical health during the period of deep sedation, with the aim that M would wake more calmly and transfer to a psychiatric ward as soon as she was physically fit. They felt that care by mental health nurses would have been beneficial when she was awake; the staff providing 1 : 1 or 2 : 1 support were often not mental health-trained.

LP struggled to obtain a psychiatric bed suitable for discharging M to. The severe psychiatric problems posed major challenges for physical health wards, and the level of physical illness proved too much for psychiatric wards. This also made rescinding her detention under the MHA extremely difficult to justify, especially when the risk of imminent death from her medical complications, were she allowed to leave the hospital, was taken into account.

### 4.5. Patient

M reported that the medical admission was an incredibly stressful time and had led to severe anxieties around returning to hospital for further appointments. Her memory of events was only partial. When asked about the positive aspects of her time in hospital, she recalled examples when staff had “bent over backwards” to help her with matters outside of the medical context, including arranging for her to leave the ward to get her hair cut and being brought ice cream by an ICU nurse whilst a tracheostomy was in place. This had a positive impact. Her recollection of the staff she liked revolved around the way they treated her as a person and continued to be friendly to her.

M said that she “hated” the restraining techniques of mittens and splints, which were used in an attempt to stop her harming herself, and they only fuelled her anxiety. She felt “trapped” and “terrified that my hands were going to fall off.” She felt that having someone to speak to about how she was feeling would have been helpful, as nobody seemed to be listening. Another episode that has led to subsequent recurrent nightmares was the use of active cooling in ICU when she was hyperthermic. She remembers being aware of being incredibly cold, but was not sure what was happening, which “terrified” her.

When asked for alternatives to manage her behaviour, she suggested bandaging up her wounds in the community and having mental health support to help her through the acute crisis. She did express a wish to be taken to hospital and staff persuading her to have treatment if things became more complicated and an infection developed. She felt that the management of her self-harm during her time on Springbank, where restraint was never used, was far more helpful.

## 5. Discussion

The complexity of this case highlights important clinical issues of everyday practice:

1. Whether people retain the capacity to make decisions about their care in a crisis
2. The medium- and long-term complications that can arise from compulsory treatment in patients who harm themselves or are suicidal in the context of a personality disorder
3. The difficulties of managing patients with major psychiatric illnesses in medical settings and vice versa

These issues arise in patients with less severe personality disorders. Every ED will have a group of patients who present frequently and in similar circumstances. One of the reasons for the high number of complications, in this case, was because of the remarkable tolerance the patient had for sedative medication, which led to an escalation in interventions that culminated in ICU admission. It is possible that the patient's long history of exposure to psychotropics led to the development of this tolerance.

Despite her severe illness, significantly fewer complications occurred when restrictive measures were avoided, and the patient was treated as having the capacity to make decisions about her care and being allowed to do so. The change in M's medication was crucial for her improvement, but this required her to be concordant, even after discharge. The ward's environment provided the opportunity for her to develop trust in the staff and her treatment plan, which stopped the historical patterns of nonconcordance, deterioration, and readmission to hospital after discharge.

In cases like this, the criteria for detention under the MHA will usually be met. Therefore, the debate is whether it is necessary and appropriate to make a recommendation for detention when they first present to services. This requires multiple considerations, including weighing the benefits against the adverse effects of detention, assessing the capacity of the patient, and thinking about the best interests [13]. The evidence provided here supports the argument that the benefits of the least restrictive approach are significant, even in such extreme circumstances. Likewise, the adverse effects of detention may be far worse than the presenting problem.

Holding someone with a severe mental disorder in an acute medical environment using police and handcuffs sets up a difficult situation from the outset. To avoid detention on admission, emergency departments require support from liaison psychiatry teams, as ED clinicians will always err on the side of caution to prevent someone from leaving and completing suicide. ED and liaison psychiatry staff should be working very closely to formulate bespoke management plans for patients who frequently attend and reenact the cycle of refusal of help and coercive care. Such plans should also be shared across the entire local acute services concordat (ambulance, police, mental health, and acute hospitals), in order to avoid coercion at the first presentation. Unfortunately, M did not have such a plan in place.

## 6. Conclusion

This case demonstrates the difficulty of making decisions about capacity and detention under the MHA for a patient with a personality disorder, chronic suicidality, and regular self-harm. The patient identified being listened to and persuaded, rather than restrained, as the most helpful intervention in a crisis. When persuasion did not work, coercion was detrimental. The most important specialist interventions needed were time, verbal deescalation, and a good therapeutic relationship. Once compulsory treatment begins in the ED, the complications that may arise make it much more difficult for other hospital settings to adopt a least-restrictive approach. The management of such patients when they first present to services is critical.

There is an urgent need for the routine evaluation of the outcomes of compulsory and noncompulsory approaches in the management of chronic suicidality and self-harm in people suffering from a personality disorder. We hypothesise that having a much higher threshold for compulsory interventions, as well as assuming that patients have capacity and respecting their autonomy, will reduce costs, yield better outcomes, and be preferred by clinicians and patients. Bespoke management plans for frequent attenders which are shared across the system could help enable this.

## Figures and Tables

**Table 1:** Summary of interventions and complications during the acute hospital admission. There were at least 17 episodes of physical restraint, with nine requiring rapid tranquillisation and four requiring intubation and admission to ICU.

Day	Intervention and treatment	Complications or unintended consequences
1	Restraint, rapid tranquillisation, high-dose antipsychotics and benzodiazepines (above British National Formulary (BNF) maximum dose) Dressed wounds	Transfer to ICU for intensive monitoring

2	Restraint, rapid tranquillisation, above BNF maximum dose antipsychotics and benzodiazepines	

3		Clinical deterioration and signs of neuroleptic malignant syndrome (NMS) were noted, with temperature of 40.6°C and creatinine kinase (CK)>5000 units/L

5	Restraint and sedation with midazolam and propofol PICC line insertion	

12		Clostridium difficile diarrhoea, acute kidney injury

13	Nasogastric (NG) tube	

23	Restraint and rapid tranquillisation twice	Sepsis, acute kidney injury, possible neuroleptic malignant syndrome

27		methicillin-resistant Staphylococcus aureus (MRSA) septicaemia confirmed
35	Restraint and rapid tranquillisation followed by sedation and intubation Wound debrided	Left internal jugular vein thrombus

48	Tracheostomy	Left lower lobe collapse, pneumothorax

50		Superior ventricular tachycardia, septic shock secondary to chest infection

52	Bronchoscopy	Hypotension unresponsive to inotropes, anaemia, blood transfusion

53	Active cooling, continuous veno-venous hemodiafiltration (CVVHDF)	Patient reports memories of waking up during active cooling, nightmares of being covered with ice

55		*Klebsiella* sepsis, ongoing renal failure, herpes simplex virus (HSV) isolated from bronchoscopy

62		Pulmonary embolus and pulmonary infarction, right heart strain

71		Fungal septicaemia

94	Restrained and sedated	

99	Subcutaneous fluids, NG feeding, catheterisation, redetention under S3 MHA	

103	2 : 1 observations	Urinary tract infection

108	Restraint and rapid tranquillisation twice	

116	Restrained and rapid tranquillisation failed, reintubation and transfer to ICU Arterial and central venous line inserted	

120	Restraint, mitts and splints put on patient to prevent self-harm	Stridor requiring adrenaline and nebulisers

123	Restrained and sedated	

128	Transferred to specialist unit	

ICU: intensive care unit.

**Table 2:** Summary of incidents and interventions during Springbank admission. There were no major complications from any intervention (or lack of intervention), apart from the pain, bleeding, and scarring from the wounds and limited skin infections that responded to antibiotics. A&E refers to the accident and emergency department.

Significant events	Day	Incident description	Intervention
Admission	1	Scratching an old wound on her left leg, making it bleed	Asked patient to stop and to redress wound.

	4	Picking at a wound on her leg until bleeding slightly, 2 inches in length	Refused to have wound cleaned, but accepted dressing. 1 : 1 support provided.

	16	Reopened wound on lower left leg using fingers	Wound cleaned and dressed. 1 : 1 support provided.

Discharged from s3 MHA	18	Three-inch laceration to her left forearm using a pair of nail scissors	Wound cleaned and dressed. 1 : 1 support provided.

	24	Inserted finger into old wound on left lower leg and then inserted an implement from nail kit into wound (7-9 cm long, inserted half-way)	Verbal support provided. Duty doctor called. Wound cleaned and dressed.

	47	Superficial scratches to face and left forearm	No intervention needed

	53	Cut with a piece of ceramic	Wound cleaned and dressed. 1 : 1 support provided. Safe held whilst cleaning.

	53	Self-harmed after smashing a ceramic bowl (cut 5 cm long and 2 mm wide)	Refused all first aid. Eventually allowed gauze and dressings, but no steri-strips.

	53	Inserted a wooden stick (2 inches long) into a wound on her arm	Duty doctor called. Refused to go to A&E and refused to have wound cleaned. Accepted dressings.

	74	Patient re-opened old wound on left arm	Wound cleaned, dry dressing applied.

	116	Self-harmed using aerosol spray. One-inch burn in her arm	Refused to see duty doctor. Advised to clean skin.

	116	Further self-harm using aerosol spray. Small red area on forearm	PRN medication and verbal support.

	205	Verbal altercation with another patient. M was punched on the nose by the other patient. No fracture	Staff support and safeguarding raised.

	224	Whilst on leave to supported accommodation, left property and told staff she was going to the motorway to end her life because she was not able to cope with the voices	Police informed. Patient called repeatedly by staff. Initially refused, but eventually agreed to return to the ward. No police involvement needed.

	237	Mania-like behaviour (poor sleep, overspending, and elation) with auditory hallucinations and paranoia. Requesting to leave the ward to jump in front of traffic as instructed by voices. Detained under s5(4), then s5(2) of the MHA. Series of incidents followed. Smashed a bowl, made multiple lacerations on her arm in the dining room in front of staff (up to 6 inches long and 1-inch deep, fatty tissue visible). Began inserting pen into laceration wounds	Staff verbally de-escalated, but did not stop her. Eventually allowed steri-stripping, but refused A&E.

	237	Inserted a pen and a hair clip into her arm through wound made earlier. Inserted on the horizontal plane through fatty tissue	Refused to go to A&E and to let staff remove objects. Unable to close wound. Dressed with gauze and bandages.

Detained s5(2) MHA	237	Reopened wound on the right arm. Inserted a piece of emery board	Refused steri-strips and A&E. Allowed cleaning with saline.

Detained s3 MHA	238	Removed dressing and inserted fingers into wound. Some pooling of blood on the floor	Allowed steri-strips only.

	238	Reopened wound on the left forearm and smeared blood on the wall	Refused steri-strips. Refused physical observations.

	238	Inserted plastic foreign bodies into cuts on left arm. Sprayed aerosol into wounds	Refused to have wounds cleaned, verbal deescalation, wounds dressed. Refusing medication and antibiotics.

	239	Smashed a glass and made 3 lacerations. One laceration made to the upper left arm, 13 cm in length, 4-5 cm wide. One laceration made to the left wrist, 6-7 cm in length. One laceration made to the left leg	Refused A&E, refused medical review, minimal first aid allowed by patient.

Second opinion from a psychosis specialist Discharged from s3 MHA	239	Made three incisions to her right bicep. Two cuts 7 cm in length 3 cm wide, one cut 10 cm in length 3 cm wide. All cuts showing fatty tissue	Refused A&E and wound dressings, but then allowed dressings. Allowed removal of plastic foreign bodies inserted before. Second opinion sought after incident. Discharged from section 3 MHA. Patient asked to leave ward. Backed down when allowed. Allowed removal of foreign bodies. Began accepting medication and wound care.

	244	Used pen to reopen lacerations to her right arm and left leg and inserted pen into her arm	Refused A&E. Allowed dressings only. Accepted PRN medication.

	247	Reopened wound on the left upper arm	Refused all interventions and 1 : 1 support. Accepted cleaning and dressing of wounds.

	247	Inserted a wooden skewer into the wound on her left forearm	Refused A&E, unable to extract foreign body, wound dressed.

	258	Inserted wooden cocktail stick into arm wound from previous self-harm	Agreed to attend A&E the following morning for removal of foreign body.

Discharged from hospital	372		

## Supplementary Materials

Supplementary Materials Tables 3 and 4 provide further information about M's hospital stays. Table 3 reflects her mental health progress since admission to Springbank using multiple validated outcome measures at admission, 6 months into admission, at discharge, and 6 months following discharge. Table 4 gives an overview of M's service use after her admission to Springbank ward using multiple measures, including emergency department presentations and admissions to psychiatric wards [14–21].
