# Supplementary material for: Iatrogenic Complications of Compulsory Treatment in a Patient Presenting with an Emotionally Unstable Personality Disorder and Self-Harm
Source: Case Rep Psychiatry. 2021 May 27;2021:6615723. doi: 10.1155/2021/6615723 (PMC8175160; doi:10.1155/2021/6615723)
Supplement: Supplementary Materials — Tables 3 and 4 provide further information about M's hospital stays. Table 3 reflects her mental health progress since admission to Springbank using multiple validated outcome measures at admission, 6 months into admission, at discharge, and 6 months following discharge. Table 4 gives an overview of M's service use after her admission to Springbank ward using multiple measures, including emergency department presentations and admissions to psychiatric wards [14–21]. [file 6615723.f1.docx]

**Supplementary Data**

| Measure | Subscale | Admission to Springbank | 6 months | Discharge | 6 months after discharge | Change at discharge compared to admission | Change at 18 months after discharge |
| --- | --- | --- | --- | --- | --- | --- | --- |
| *RFL [14]* | Survival Coping Beliefs | 1.3 | 4.2 | 4.5 | 4.7 | Improved | Improved |
|  | Responsibility to Family | 3.6 | 5.1 | 5 | 4.7 | Improved | Improved |
|  | Child related concerns | 1 | 1 | 1 | 1 | No change | No change |
|  | Fear of suicide | 3.7 | 4.4 | 2.7 | 3.4 | Deteriorated | Improved |
|  | Fear of social disapproval | 5 | 4.3 | 4.3 | 4.7 | Deteriorated | Improved |
|  | Moral objection | 1.5 | 2.5 | 1.5 | 2.5 | No change | Improved |
|  | ***Mean*** | **2.2** | **4** | **3.9** | **4.1** | Improved | Improved |
| *CORE [15]* | Well-Being | 4 | 2.75 | 2 | 2.5 | Improved | Improved |
|  | Symptoms | 4 | 1.83 | 2.75 | 3.3 | Improved | Improved |
|  | Functioning | 3.33 | 2.17 | 1.75 | 1.8 | Improved | Improved |
|  | Risk | 3.67 | 0.17 | 1.67 | 1.2 | Improved | Improved |
|  | Non-Risk | 3.71 | 2.11 | 2.21 | 2.5 | Improved | Improved |
|  | ***Mean*** | **3.7** | **1.8** | **2.1** | **2.3** | Improved | Improved |
| *GAD-7 [16]* | Total | **13** | **18** | **21** | **16** | Deteriorated | Improved |
| *DERS [17]* | Non-accept | 30 | 26 | 24 | 29 | Improved | Improved |
|  | Goals | 24 | 11 | 19 | 21 | Improved | Improved |
|  | Impulse | 29 | 12 | 13 | 15 | Improved | Improved |
|  | Awareness | 19 | 16 | 19 | 19 | No change | No change |
|  | Strategies | 39 | 17 | 17 | 25 | Improved | Improved |
|  | Clarity | 17 | 10 | 16 | 14 | Improved | Deteriorated |
|  | ***Mean*** | **158** | **92** | **108** | **123** | Improved | Improved |
| *KIMS [18]* | Observe | 12 | 31 | 27 | 34 | Improved | Improved |
|  | Describe | 8 | 23 | 24 | 19 | Improved | Improved |
|  | Act with awareness | 10 | 29 | 29 | 32 | Improved | Improved |
|  | Accept without judgement | 9 | 40 | 31 | 41 | Improved | Improved |
|  | ***Mean*** | **39** | **123** | **111** | **126** | Improved | Improved |
| *QPR [19]* | Interpersonal | 0 | 12 | 52 | 35 | Improved | Improved |
|  | Intrapersonal | 0 | 4 | 18 | 15 | Improved | Improved |
|  | ***Mean*** | **0** | **16** | **70** | **50** | Improved | Improved |
| *SWEMWBS [20]* | ***Total*** | **7** | **21** | **31** | **20** | Improved | Improved |
| *EQ5-D [21]* | ***Total*** | **-0.285** | **0.662** | **0.816** | **1** | Improved | Improved |

# **Table 3:** Results of E’s structured outcome measures at Springbank Ward. Abbreviations: RFL (Reasons For Living), CORE (Clinical Outcomes Routine Evaluation), GAD-7 (Generalised Anxiety Disorder 7), DERS (Difficulties in Emotion Regulation Scale), KIMS (Kentucky Inventory of Mindfulness Skills), QPR (Questionnaire about the Process of Recovery), SWEMWBS (Short Warwick–Edinburgh Mental Well-Being Scale), EQ5-D (EuroQol health related quality of life score)

| Service use over 248 days | Before admission | After admission |
| --- | --- | --- |
| Presentations to the Emergency Department | 5 | 0 |
| Admissions to acute psychiatric wards | 14 | 0 |
| Detentions under a section of the Mental Health Act | 3 | 0 |
| Number of electronic record entries | 584 | 141 |
| Electronic records word count | 79,618 | 20,666 |

**Table 4.** Service use before and after admission to Springbank. *At the time of writing, M has been discharged for 248 days, so this was the period used for the comparison.*
